# Real-Time Modeling of Volume and Form Dependent Nanoparticle Fractionation in Tubular Centrifuges

**DOI:** 10.3390/nano12183161

**Published:** 2022-09-12

**Authors:** Marvin Winkler, Frank Rhein, Hermann Nirschl, Marco Gleiss

**Affiliations:** Institute of Mechanical Process Engineering and Mechanics, Karlsruhe Institute of Technology (KIT), 76131 Karlsruhe, Germany

**Keywords:** solid–liquid separation, fractionation, tubular centrifuges, dynamic modeling, real-time simulation, multi-dimensional particle properties

## Abstract

A dynamic process model for the simulation of nanoparticle fractionation in tubular centrifuges is presented. Established state-of-the-art methods are further developed to incorporate multi-dimensional particle properties (traits). The separation outcome is quantified based on a discrete distribution of particle volume, elongation and flatness. The simulation algorithm solves a mass balance between interconnected compartments which represent the separation zone. Grade efficiencies are calculated by a short-cut model involving material functions and higher dimensional particle trait distributions. For the one dimensional classification of fumed silica nanoparticles, the numerical solution is validated experimentally. A creation and characterization of a virtual particle system provides an additional three dimensional input dataset. Following a three dimensional fractionation case study, the tubular centrifuge model underlines the fact that a precise fractionation according to particle form is extremely difficult. In light of this, the paper discusses particle elongation and flatness as impacting traits during fractionation in tubular centrifuges. Furthermore, communications on separation performance and outcome are possible and facilitated by the three dimensional visualization of grade efficiency data. Future research in nanoparticle characterization will further enhance the models use in real-time separation process simulation.

## 1. Introduction

Processing and purification of nanoscale products is becoming increasingly important in modern industry and science. One of the main reasons lies in the fact that nowadays, the connection between specific nanoparticle (NP) traits and general improvements of key product properties are well known [1,2,3,4,5,6,7,8]. Unit-operations regarding bottom-up synthesis [9,10,11] as well as top down formulation procedures [12,13,14] are therefore tailored towards high yields of narrowly distributed and beneficial particle sizes, shapes or material compositions. In many cases the use of additional separation steps to sort nanoparticles and remove impurities are mandatory [15,16]. In light of this, the demand for a multi-dimensional separation- and grade efficiency quantification in particle technology is increasing [17,18]. This means that several geometric and material parameters of a particle collective have to be evaluated simultaneously [19,20,21]. Following this goal, initial affords expand traditional schemes such as one dimensional (1D) particle size distributions (PSD) to e.g., two dimensional (2D) property distributions [22,23]. To clarify the notation used in this paper, the concept of particle trait distribution (PTD) is introduced. Traditional PSDs are identical to 1D-PTD because only one feature is highlighted. The consideration of multiple particle properties lead to higher dimensional PTDs, e.g., a three dimensional (3D) PTD.

In the field of solid–liquid separation, tubular bowl centrifuges are capable of processing nanosuspensions on a bench-scale with high throughput rates. Due to high centrifugal forces the apparatus can address challenging fractionation tasks of ultrafine particles and biological products [24,25,26,27,28,29]. The evaluation of the apparatus performance is based on the physical properties of a fine (centrate) and coarse fraction (sediment). Monitored process information usually include the solids mass fraction and a grade efficiency according to one major separation criterion (e.g., particle size). In an effort to include an additional criterion, Winkler et al. [30] studied the simultaneous fractionation according to particle size and solids density of mixed nanosuspensions experimentally. Moreover, the authors established a real-time monitoring methodology to evaluate the fine fraction composition [31]. The latter is necessary because tubular bowl centrifuges operate semi-continuously. The reason for this is that the accumulated sediment affects the separation result at longer process times. The experimental studies listed in this paragraph show that tubular bowl centrifuges can be successfully used for the fractionation of submicron- and nanosuspensions. However, they also underline that a large number of experiments, online monitoring and laboratory analyses are necessary to reliably characterize influencing variables.

To help understand the physical behavior within the centrifuge and gather vital process knowledge efficiently, mathematical modeling is important. Build upon the idea of flowsheet simulation [32,33], recent efforts established numerical models for time-dependent separation efficiency and sediment build-up computations in solid bowl centrifuges. These so called dynamic short-cut models describe complex physical conditions during the separation process on the basis of empirical equations and model assumptions. In contrast to a macroscopic process-chain consideration, these meso-scale models incorporate apparatus geometries and specific material functions to characterize the nanosuspension behavior. The well-documented methods of Gleiss et al. and Menesklou et al. [34,35,36,37,38,39] serve as the basis for a novel tubular centrifuge model (TCM) developed in this work. Regardless of the type and implementation of the process model, they share the same advantages. They can be used in combination with experimental data to optimize individual unit operations by identifying advantageous process parameters. Moreover, they reduce experimental efforts and assist in the design of experiments. Lastly, compared to simulations based on Computational Fluid Dynamics (CFD) and the Discrete Element Method (DEM), the dynamic process models demand a low investment in computational power. Consequently, this leads to a possible application in model predictive control strategies that demand real-time process simulation speeds [40].

One commonality of the highlighted studies is the exclusive focus on particle size as the major classification characteristic. In other words, solids were assumed to be perfect spheres, resulting in 1D grade efficiency curves. Revisiting the novel concept of multi-dimensional separation- and grade efficiency quantification stresses the point that existing short-cut models for tubular bowl centrifuges should be expanded to address e.g., form dependent fractionation. The presented study aims to merge the concept of form dependent sedimentation and multi-dimensional trait distributions with the short-cut modeling of NP fractionation in tubular bowl centrifuges. The adapted simulation tool is able to process both 1D and higher dimensional PTDs as a model input. Incorporating material functions and operating parameters, the spatial and time-dependent separation of NPs during centrifugation is computed.

This article is organized as follows: Section 2 outlines the theoretical background on form dependent NP sedimentation in tubular bowl centrifuges. Additionally, mathematical formulations of 3D joint distribution density functions and their application as PTDs are described. In Section 3, vital information on the TCM implementation, its working assumptions and parameters are presented. In a first simulation case, a 1D classification setup is used to validate the TCM functionality and output (Section 4.2) with experimental data. Here, commercially available fumed silicon dioxide (SiO${}_{2}$) NPs serve as a real-world particle system (RPS). The novel approach of 3D separation efficiency modeling is tested with the help of virtually generated SiO${}_{2}$ particles, further abbreviated as virtual particle system (VPS). Their creation and statistical evaluation according to particle form and volume is described in Section 3.4. The VPS and its 3D-PTD serves as new input for a second TCM simulation case. Material parameters and functions from the 1D case are reused. Based on the model output for 3D grade efficiencies, the new TCM approach is discussed thoroughly in Section 4.

## 2. Theory

### 2.1. Characterization of Particle Shape Based on Inertia Ellipsoids

The most frequently used isotropic quantity to describe a solid particle of arbitrary shape is the so called equivalent diameter,

(1) $$d=\sqrt[{3}]{\frac{6}{\pi }V_{P}},$$

of a sphere with identical volume $V_{P}$. While it provides no morphological information, it can easily convey the particle mass if an affiliated solid density $\rho_{p}$ is given. The particles morphology, however, can differ greatly in its degree of complexity. Both smooth, convex shapes such as spheres, plates, cuboids and ellipsoids as well as more complex aggregated structures and rough surfaces are possible. An extensive overview of different form parameters found in the literature is listed in [41]. Barrett et al. [42] mentions the three independent parameters form, roundness and surface texture one may consider for describing particle shape. Here, particle form is the easiest to express and measure since almost any given form parameter is estimated based on the longest $x_{L}$, intermediate $x_{I}$ and shortest $x_{S}$ axis length. As shown in Zingg’s diagram [43], the two independent axis ratios of elongation,

(2) $$e=x_{I}\;/\;x_{L}$$

and flatness

(3) $$f=x_{S}\;/\;x_{I},$$

can express and visualize shape on a macroscopic level but exclude information on roundness and surface texture. Recent studies expanded this classification system and described a more detailed strategy to index non-spherical particles based on their elongation and flatness [44] differentiating between compact, bladed, elongated and flat objects.

**Figure 1:**
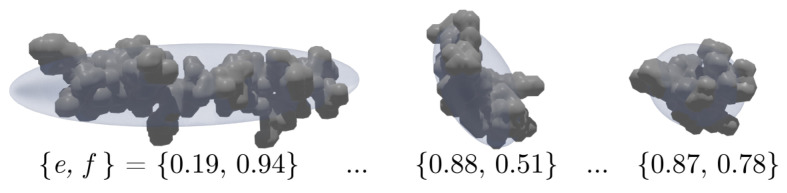
Image of three different three dimensional (3D) aggregates and their corresponding inertia ellipsoid illustrated as a transparent hull. Elongation *e* and flatness *f* values for each ellipsoid are displayed below each body.

In the scope of this work, the model strategy involves a simplification of any complex particle morphology based on a surrounding convex hull, hereby named Legendre${}^{\prime }$s ellipsoid. Rooted in the field of mechanics, it is also referred to as the inertia ellipsoid of a given compact body sharing the same moments of inertia and center of mass [45,46]. Figure 1 shows a 2D view of three complex 3D particle aggregates. Following the introduced classification criteria by Barrett et al. [42], their surface may be considered smooth, however, their overall structure is irregular and concave. The schematic also illustrates all associated inertia ellipsoids whose calculation is described in Section 3. From left to right, the first particle is elongated, the second represented by an oblate ellipsoid, and the one on the right is very compact. Throughout this paper, these scalene hulls and their axis lengths $x_{L}$, $x_{I}$ and $x_{S}$ represent the global form of any given particle.

### 2.2. Form Dependent Particle Settling in a Centrifugal Field

Along the radial axis of rotation, a smooth particle in creeping motion within a liquid medium inside a centrifuge experiences mass and frictional forces. Assuming uncharged particles in an infinitely diluted suspension with no particle-particle interactions and negligible particle diffusion, the state of force equilibrium between drag, buoyancy, and centrifugal force yields

(4) $$\begin{array}{c} \left(\frac{dr}{dt}\right)=u_{0}=\sqrt{\frac{V_{P}\;r\omega^{2}\;\Delta \;\rho }{c_{D}\;A\frac{1}{2}\;\rho_{l}}}. \end{array}$$

Equation (4) provides an expression for the terminal settling velocity $u_{0}$ of a single particle with volume $V_{p}$. Here, *r* defines the distance from the axis of rotation, ω is the constant angular velocity inside a stationary liquid pond and *t* stands for the elapsed time. Equation (4) describes the particle velocity as a function of the density difference between fluid and solid $\Delta \rho =\rho_{p}-\rho_{l}$ and the area *A* projected by the particles surface in the direction of radial flow. Lastly, the drag coefficient $c_{D}$ is a dimensionless parameter which quantifies the solids hydrodynamic drag. Regarding the assumptions made to introduce Equation (4), the influencing factors of this parameter include the particle Reynolds Number,

(5) Rep=ρldu0ηl,

geometric shape and orientation. Here, *d* is introduced as the equivalent diameter of a volume-equivalent sphere and $\eta_{l}$ the dynamic viscosity of the fluid. Due to the small length-scales of colloids, a creeping motion with low Reynolds numbers Rep≪1 can be expected. In this regime, Stokes [47] found an analytical solution for spherical bodies with the definition cD,S=24/Rep for the drag coefficient, using Equation (1) and the projection area $A=\frac{\pi }{4}\;d^{2}$. Substituting these expressions in Equation (4) leads to the well-known expression of the Stokes settling velocity

(6) $$\begin{array}{c} u_{0,S}=\frac{d^{2}\;\Delta \rho \;r\;\omega^{2}}{18\eta_{l}}, \end{array}$$

for a spherical particle. In terms of particle shape, Equation (6) describes sedimentation in one dimension. The consideration of multiple geometric particle properties by drag correlations in several ranges of Reynolds numbers is extensively studied and discussed in the literature [41,48,49,50,51,52,53]. A recently published article by Trunk et al. [41] compiles multiple drag correlations for non-spherical particles and provides additional information on the drag correlation approximation for Reynolds numbers outside the Stokes regime. In the context of this work the mathematical formulation of the corrected drag coefficient,

(7) $$\begin{array}{c} c_{D}^{*}=f\left(Re_{p},k_{S}\right)=c_{D,S}\;k_{S}, \end{array}$$

is defined as the product of $c_{D,S}$ and the so called Stokes drag correction factor $k_{S}$ in accordance with the definition used by Ganser et al. [49]. This simplification is only valid in the Stokes regime but applicable for this work, since the presented model is restricted to NP sedimentation. With the identical transformation strategy that was used for Equation (6), Equations (4) and (7) yield a approximation for the terminal settling velocity of non-spherical particles:

(8) $$\begin{array}{c} u_{0}\left(k_{S}\right)=\frac{d^{2}\;\Delta \rho \;r\;\omega^{2}}{k_{S}\;18\eta_{l}}\;. \end{array}$$

Besides Stokes’ settling theory, analytical solutions for $k_{S}$ were obtained for spheroids [54] and other axisymmetric particle shapes [55,56]. The equations listed use synonyms of the form descriptors $x_{L}$, $x_{I}$ and $x_{S}$ introduced in Section 2.1. Building upon this well documented knowledge, recent studies of Bagheri et al. [53] dealt with the prediction of the translational friction coefficient in gaseous and liquid media for a wide range of sub-critical Reynolds Numbers ($Re_{p}<3\times 10^{5}$). The authors claim, that shape descriptors defining $k_{S}$ should be easy to measure and not dependent on the complex determination of e.g., sphericity in 3D scenarios. Consequently, they are defined by form- rather than roundness- or surface texture parameters which suits the purpose of this study. The presented definition of the drag correction factor

(9) $$k_{S,B}=\frac{1}{2}\left({\left(f\;e^{1.3}\right)}^{\alpha }+{\left(f\;e^{1.3}\right)}^{-\alpha }\right)$$

is merely a function of the particles elongation and flatness. The exponent α is set to 1/3 following the assumption that particles tend to settle with neither a preferred orientation but rather with a random rotation in the Stokes regime [53]. Migration due to Brownian motion is common for NPs [57,58] which explains this assumption. Hence, Equation (9) is applicable when approximating the NP settling velocity in tubular bowl centrifuges. Following the model theory, the drag correction factor for strongly elongated and flat particles is high. Substituting $k_{S,B}$ in Equation (8) with α=1/3 yields

(10) $$u_{0,B}=\frac{d^{2}\;\Delta \rho \;}{6\;\eta_{l}\left({\left(f\;e^{1.3}\right)}^{1\;/\;3}+{\left(f\;e^{1.3}\right)}^{-1\;/\;3}\right)}\;r\;\omega^{2}=s_{0,B}\;r\;\omega^{2},$$

for the sedimentation rate of arbitrary shaped, randomly oriented particles in creeping motion. Its diameter *d* and density define the mass of an equivalent spherical body while its shape induced deceleration is approximated by a scalene inertia ellipsoid with elongation *e* and flatness *f*. Additionally, Equation (10) introduces the sedimentation coefficient $s_{0,B}$ as a particle trait dependent scalar with unit $s^{-1}$. Consequently, this parameter is dependent on the physical properties of both the solid and the surrounding fluid as well as the particle shape.

The presented expressions for the sedimentation coefficient are valid for highly diluted suspensions which have low technical relevance. Both density and viscosity of the suspension increase with increasing solids volume fraction. In addition, a solvent backflow is generated by the moving particles [59]. At high dilution, these hydrodynamic interactions induce a first order dependence of particle friction on the solids volume fraction ϕ [60,61]. As a consequence an expression for the apparent sedimentation coefficient,

(11) $$\begin{array}{c} s_{B}=s_{0,B}\;{\left(1+w\;\phi \right)}^{-1}, \end{array}$$

may be defined, where *w* is the hindered settling coefficient. For hard spheres, empirical observations are reported in the literature describing the influence of concentration on the settling behavior of mono- and polydisperse suspensions [62,63,64]. In the case of non-isometric particles shapes, only few experimental studies are reported in the literature. There are studies that have investigated the settling behavior of variously shaped macro-molecules or colloidal platelets. Again a linear relationship between the concentration and the normalized sedimentation velocity was found for low solid volume fractions [65]. Particularly noteworthy is the increase in particle resistance due to the decreasing sphericity and the resulting increased backflow of the solvent. Such results emphasize that the influence of concentration may not be neglected neither in experimental studies, nor in any model approach of colloidal particle sedimentation.

### 2.3. Joint Particle Trait Distributions

In particle technology, probability density distributions or PTD in general are used both for the characterization of a particulate product as well as for the quantification of a separation experiment outcome. In most cases, a PSD is derived either from the indirect analysis of a physical property such as the attenuation or scattering of light [66,67], by sieving, or by direct image processing [68,69]. Here, the equivalent particle diameter *d* is commonly used as a random variable $Z=\{d_{1},d_{2},...,d_{M}\}$, representing the arithmetic mean of each discrete particle size class in a predefined sample space. The distribution weighting is defined by the subscript r. The probability *P* of a particle occurrence in interval $\left[d_{1},d_{2}\right]$ is defined by:

(12) $$\begin{array}{c} P\left(d_{1}\leq Z\leq d_{2}\right)=\int_{d_{1}}^{d_{2}}q_{r}\left(d\right)\;dd, \end{array}$$

for *Z* being a continuous random variable. In probability theory, $q_{r}\left(d\right):R\to R$ is defined as a probability density function of *Z* which has to satisfy $q_{r}\left(d\right)\geq 0$ and $\int_{-\infty }^{\infty }q_{r}\left(d\right)\;dd=1$. However, a dynamic process model for separation based on arbitrary shaped particles and their respective inertia ellipsoids calls for at least two random variables. Let *Y* be a second discrete random variable that takes on values $\left\{e_{1},e_{2},...,e_{L}\right\}$ and *X* be a third discrete random variable that takes on values $\left\{f_{1},f_{2},...,f_{K}\right\}$ representing the form parameters elongation and flatness. Then, the joint probability density function of the continuous random vector (Z,Y,X) is $q_{r}\left(f,e,d\right):R^{3}\to R$. The situation is analogous to the 1D case where the probability to meet a particle in a given interval,

(13) $$\begin{array}{c} P\left(f_{1}\leq X\leq f_{2},\;e_{1}\leq Y\leq e_{2},\;d_{1}\leq Z\leq d_{2}\right)=\int_{f_{1}}^{f_{2}}\int_{e_{1}}^{e_{2}}\int_{d_{1}}^{d_{2}}q_{r}\left(f,e,d\right)\;df\;de\;dd, \end{array}$$

is equal to the volume of an infinitesimal cuboid of length df, width de and height dd times the probability density at (f,e,d). Again, $q_{r}\left(f,e,d\right)\geq 0$ and

(14) $$\begin{array}{c} \int_{-\infty }^{\infty }\int_{-\infty }^{\infty }\int_{-\infty }^{\infty }q_{r}\left(f,e,d\right)\;df\;de\;dd=1 \end{array}$$

must hold [70,71]. This means that for a particle trait distribution to be normalized neither the area nor the volume under a curve or plane must equal unity, but rather the sum of a four dimensional hyperspace. In Section 4, this is of interest for visualizing a particle trait distribution with three defining geometric variables.

Equations (12) and (13) can be formulated analogously as discrete distributions with sums of individual property classes, which are better suited for applications in separation technology. This is because numeric calculations involving PTDs are usually formulated in a discrete manner. In addition, experimentally determined distributions are given in discrete values, which result in an adequate histogram. In the scope of this work, volume weighted distributions are used for both particle trait distribution visualization and calculations in the presented dynamic process model for tubular bowl centrifuges.

One major difficulty associated with 3D distributions is the fact that their representations are often convoluted and lack clarity. To overcome these challenges in data visualization, marginalization is an adequate tool to reduce the dimensional complexity and potentially enhance the readability of higher dimensional data. In the following, the mathematical computation needed for the marginalization of 3D data to 2D and 1D datasets is shown. Referring to strategies used throughout this study we define:

(15) $$\begin{array}{c} q_{3}\left(f,e\right)=\int_{d_{\min}}^{d_{\max}}q_{3}\left(f,e,d\right)\;dd, \end{array}$$

as the marginalized volume weighted distribution density by integration over the equivalent particle diameter *d*. Analogously, performing a two step integration over elongation *e* and flatness *f*, a marginalized distribution density,

(16) $$\begin{array}{c} q_{3}\left(d\right)=\int_{f_{\min}}^{f_{\max}}\int_{e_{\min}}^{e_{\max}}q_{3}\left(f,e,d\right)\;df\;de, \end{array}$$

in one dimension can be derived. The method of marginalization is used in Section 4 for discussion and data visualization purposes.

## 3. Materials and Methods

This section introduces the tubular bowl centrifuge used in both experimental and numerical investigations. Furthermore, the dynamic simulation tool is thoroughly described and all necessary balance equations and model assumptions regarding the time dependent NP separation are given. It builds upon the work of Gleiss [72] and published work of his research group at the IMVM in Karlsruhe. Lastly, physical information on two SiO${}_{2}$ NP suspensions are given. These particle systems are used to validate the 1D model output and help to understand the challenges in 3D and multi-dimensional separation setups.

### 3.1. Tubular Centrifuge Design Equations

**Figure 2:**
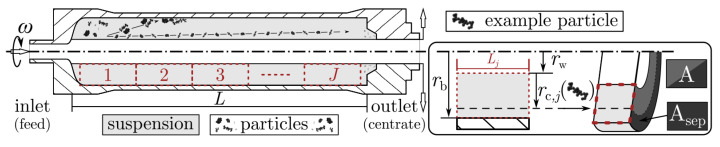
Schematic cross section of a tubular centrifuge rotor highlighting the geometry and spatial discretization with j={1,2,⋯,J} cylindrical compartments of equal volume.

This section deals with the mathematical description of the tubular centrifuge design equations used to model the separation process regarding single particles in motion. Figure 2 depicts a schematic cross section of a tubular centrifuge rotor. Typically, in most commercially available devices, the rotor is mounted in a suspended position and accelerated to a desired angular velocity ω. The second important operating parameter is the volume flow that forces the suspension through the vessels bottom inlet. During operation, the centrifugal force forms a liquid pond, the height of which is set by the rotor weir at the outlet. Additionally, a gaseous core centered around the rotor axis is present. The exemplified trajectory in Figure 2 of particles with different sizes visualizes the apparatus general classification use. Coarser fractions accumulate at the rotor wall whereas smaller particles are transported beyond the overflow weir and enter the centrifuge downstream. Depending on the initial feed concentration, this build-up sediment can quickly reduce the available liquid cross section and diminish the separation efficiency over time.

Abstracted in two dimensions, the particles settling path in the fluid reservoir is set by its radial and axial velocity. The ratio of these two velocities is decisive for the separation efficiency approximation. By assuming plug flow inside any compartment with length $L_{j}$ in the liquid pond, the particles residence time,

(17) $$\begin{array}{c} t_{v,j}=\frac{V}{\dot{V_{f}}}=\frac{\pi \left(r_{b,j}^{2}-r_{w}^{2}\right)\;L_{j}}{\dot{V_{f}}}, \end{array}$$

is altered by the liquid throughput ${\dot{V}}_{f}$ and the surface area $A=\pi \left(r_{b,j}^{2}-r_{w}^{2}\right)$ of the liquid layer. The weir $r_{w}$ and boundary $r_{b,j}$ radius as well as the total length of the settling zone *L* are set by the accumulated sediment height and the rotor geometry.

Equating the particles residence and settling time by combining Equations (10), (11) and (17) yields an expression for a critical settling position,

(18) $$\begin{array}{c} r_{c,j}=r_{b,j}\cdot \left(\exp\left(-s_{B}\cdot \omega^{2}\cdot \frac{\pi \left(r_{b,j}^{2}-r_{w}^{2}\right)\;L_{j}}{{\dot{V}}_{f}}\right)\right), \end{array}$$

for each individual particle fraction. All material and fluid characteristics influencing the particles sedimentation are comprised in the introduced model for $s_{B}$. This derivation is similar to that in publications of Gleiss et al. [35,36], who defined a grade efficiency:

(19) $$\begin{array}{c} T_{j}\left(s_{B}\right)=\frac{A_{\mathrm{sep}}}{A}=\frac{r_{b,j}^{2}-r_{c,j}^{2}}{r_{b,j}^{2}-r_{w}^{2}}, \end{array}$$

assuming a homogeneous distribution of each particle fraction along the radial axis. Here, $A_{\mathrm{sep}}$ is the area of the annular gap in which particles with an associated *s*-value will reach the rotor wall at the end of their residence time and are thus separated. As in Gleiss et al. [34] and Menesklou et al. [38], a compartment grade efficiency,

(20) $$\begin{array}{c} T_{j}\left(s_{B}\right)=T_{j}\left(d,e,f\right)=\frac{r_{b,j}^{2}}{r_{b,j}^{2}-r_{w}^{2}}\cdot \left[1-{\left(\exp\left(-s_{B}\cdot \omega^{2}\cdot \frac{\pi \left(r_{b,j}^{2}-r_{w}^{2}\right)\;L_{j}}{{\dot{V}}_{f}}\right)\right)}^{2}\right], \end{array}$$

is defined by inserting Equation (18) in (19). This expression for the grade efficiency gives an approximation for the separation probability as a function of the sedimentation coefficient, operating parameters and geometric boundaries. Additionally, this leads to a multivariate grade efficiency formulation centered at the three particle traits equivalent diameter *d* elongation *e* and flatness *f* that define $s_{B}$. The consideration of two additional dimensions requires modifications to the discretization strategy used in the transient model and balance equations described in the next section.

### 3.2. Mathematical Model and Discretization

**Figure 3:**
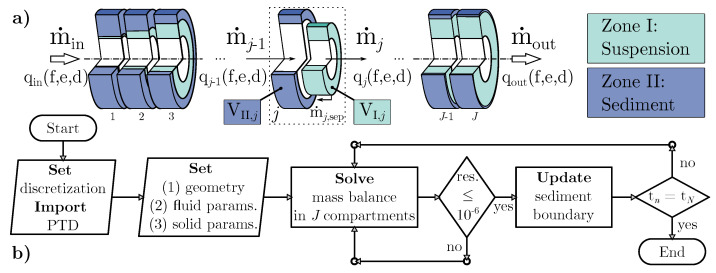
Spatial discretization of the separation zone in j={1,2,⋯,J} compartments with illustration of incoming and outgoing flows (**a**); Flow chart of the simulation algorithm (**b**).

Building on the physical processes described in Section 3.1, Figure 3 highlights the spatial discretization of the separation zone in *J* compartments of equal volume. These sections are coupled by mass balances involving both suspended solids in the suspension zone (I) and deposited material in the sediment zone (II). Consequently, both particle sedimentation and sediment build-up are calculated locally with a desired spatial resolution. By using a finite amount of compartments, the temporal change in sediment height and particle residence time are approximated with low numerical effort [35].

One resulting advantage is that this strategy allows real-time modeling of the transient separation process. Referring to the illustration of compartment *j* in Figure 3, the mass balance equation of the suspension zone,

(21) $$\begin{array}{c} \frac{dm_{I,j}}{dt}={\dot{m}}_{I,j-1}-{\dot{m}}_{I,j}-{\dot{m}}_{j,\mathrm{sep}}, \end{array}$$

describes the temporal mass change. Assuming a constant particle density, their mass can be substituted by the product of sedimentation zone volume $V_{I,j}$ and solids volume fraction $\phi_{I,j}$ resulting in the expression:

(22) $$\begin{array}{c} \frac{d(\phi_{I,j}\;V_{I,j})}{dt}=\dot{V_{f}}\;\phi_{I,j-1}\left(1-E_{j,\mathrm{sep}}\left(t\right)\right)-\dot{V_{f}}\;\phi_{I,j}\;. \end{array}$$

Here, the ratio of separated to incoming particle mass is introduced as the local separation efficiency $E_{j,\mathrm{sep}}\left(t\right)$. Analogously, the change in sediment volume $V_{\mathrm{II},j}$ over time,

(23) $$\begin{array}{c} \frac{d(\phi_{\mathrm{II},j}\;V_{\mathrm{II},j})}{dt}=E_{j,\mathrm{sep}}\left(t\right)\cdot \dot{V_{f}}\cdot \phi_{I,j-1}, \end{array}$$

is equal to the mass flow rate of the solids which do not migrate to the next compartment and are thus separated. For the calculation of $E_{j,\mathrm{sep}}\left(t\right)$ is calculated from $\dot{V_{f}}$, ω and $s_{B}$ as shown in Equation (20).

The general, discrete formulation of the separation efficiency,

(24) $$\begin{array}{cc} E_{j,\mathrm{sep}} & ={\dot{m}}_{j,\mathrm{sep}}\;{\left({\dot{m}}_{I,j-1}\right)}^{-1}=\sum_{k=0}^{K}\sum_{l=0}^{L}\sum_{m=0}^{M}\left(T_{j}\left(f_{k},e_{l},d_{m}\right)\;q_{3,j}\left(f_{k},e_{l},d_{m}\right)\right), \end{array}$$

will now be further explained based on the introduced distribution theory in Section 2.3. Given any particle collective, a discrete, mass weighted formulation of Equation (12) yields:

(25) $$m_{j}\left(d_{m}\right)=q_{3,j}\left(d_{m}\right)\;\Delta d\;\sum_{m=0}^{M}m_{j}\left(d_{m}\right),$$

for the mass fraction $m_{j}\left(d_{m}\right)$ of a particle with diameter $d_{m}$ in a class with uniform interval width Δd. *M* is the number of discrete diameter classes. Consequently, both distribution density $q_{3,j}\left(d_{m}\right)$ and compartment grade efficiency $T_{j}\left(d_{m}\right)$ (Equation (20)) are vectors with size $\left(1\times M\right)$. When incorporating two additional dimensions, the mass fraction,

(26) $$m_{j}\left(f_{k},e_{l},d_{m}\right)=q_{3,j}\left(f_{k},e_{l},d_{m}\right)\;\Delta f\;\Delta e\;\Delta d\;\sum_{k=0}^{K}\sum_{l=0}^{L}\sum_{m=0}^{M}\;m_{j}\left(f_{k},e_{l},d_{m}\right),$$

includes $q_{3,j}\left(f_{k},e_{l},d_{m}\right)$ and $T_{j}\left(f_{k},e_{l},d_{m}\right)$ as 3D arrays of shape $\left(K\times L\times M\right)$. Both in the traditional 1D case and in the extended 3D consideration, $E_{j,\mathrm{sep}}$ is calculated from a numerical integration of the product of the separation efficiency and the distribution density as shown in Equation (24). The departing PTD is linked to the one in the prior compartment $\left(j-1\right)$ or in case of j=1 to the initialized feed distribution $q_{3,\mathrm{in}}$ as shown in Equation (27).

(27) $$\begin{array}{c} q_{3,j}\left(f_{k},e_{l},d_{m}\right)=q_{3,j-1}\left(f_{k},e_{l},d_{m}\right)\frac{\left(1-T_{j}\left(f_{k},e_{l},d_{m}\right)\right)}{\left(1-E_{j,\mathrm{sep}}\right)} \end{array}$$

Consequently, Equations (22) and (23) form a linear system of equations solved by the algorithm depicted in Figure 3b. For its implementation, the python3 programming language is used. The list of mandatory repositories include the *numpy* [73] package and function *fsolve* [74,75] from the *scipy* package. At the start of a simulation run, the compartment count *J*, the initial PDT, solid and liquid parameters as well as the rotor geometry is set. The introduced system of balance equations is solved implicitly for a user defined time step size Δt and total simulation time $t_{N}$. A solution is found for each $t_{n}$ if the relative residual error between two consecutive iterates is at most $10^{-6}$. Due to the progressive reduction of the volume $V_{I,j}$, the boundary radii $r_{b,j}$ increase and the local grade efficiencies must be recalculated for each time step. The first salient model output is the centrates solids volume fraction expressed as $\phi_{\mathrm{out}}\left(t_{n}\right)$. With the known feed concentration $\phi_{\mathrm{in}}$ an expression for the global separation efficiency,

(28) $$E_{g}\left(t_{n}\right)=1-\frac{\phi_{\mathrm{out}}\left(t_{n}\right)}{\phi_{\mathrm{in}}},$$

is set. The second model output calculation centers around the global grade efficiency,

(29) $$\begin{array}{ccc} T_{g}\left(d_{m},t_{n}\right) & =1-\frac{E_{g}\left(t_{n}\right)\;q_{3,\mathrm{out}}\left(d_{m},t_{n}\right)}{q_{3,\mathrm{in}}\left(d_{m}\right)}\;\;\mathrm{and} & \;\left(1D\right) \end{array}$$

(30) $$\begin{array}{ccc} {\tilde{T}}_{g}\left(f_{k},e_{l},d_{m},t_{n}\right) & =1-\frac{{\tilde{E}}_{g}\left(t_{n}\right)\;{\tilde{q}}_{3,\mathrm{out}}\left(f_{k},e_{l},d_{m},t_{n}\right)}{{\tilde{q}}_{3,\mathrm{in}}\left(f_{k},e_{l},d_{m}\right)} & \;\left(3D\right), \end{array}$$

for one up to three dimensions following the fundamental grade efficiency descriptions found in the mechanical process engineering literature [76]. With Equations (28)–(30) each time step provides information on the actual separated mass per particle trait class based on the outgoing, mass weighted density distribution $q_{3,\mathrm{out}}$.

Regarding transparency and the discussion of model caveats in later sections, the following list of assumptions and model simplifications is noted:

(1) The flow pattern inside the centrifuge is plug flow. solid–liquid or solid-solid interactions are modeled solely in one direction by the hindered settling coefficient (Equation (11));
(2) Particles are small compared to the rotor length and weir perimeter. Fluid transport is therefore not influenced by the dispersed NPs;
(3) A pronounced radial dispersion within the liquid layer is assumed. In each compartment the incoming particle fractions are back-mixed ideally;
(4) Any formed sediment is incompressible and maintains a constant porosity
  $\left(\frac{d\phi_{\mathrm{II},j}}{dt}=0\right)$ simplifying the implicit solution of Equations (22) and (23) and reducing the computational effort;
(5) The gas-liquid interface has no influence on the separation process;
(6) The suspension is stabilized and particles retain their geometric shape and material density. Hence, no breakage or agglomeration takes place resulting in an immutable PTD both in the 1D and 3D case.

One note concerning the code structure and its execution is that both classification and form dependent fractionation can be addressed. Inside the computational domain, multiple TCM cases with different initial boundary conditions, inputs and discretization parameters can be set up. If the input PTD is a 1D-array holding discretization variable *d*, a classification is calculated and the assumption e=f=1 for each particle class holds. The second considered setup is that the input PTD represents a tensor. Here, *d*, *e* and *f* define the volume and form properties of the respective particle classes. All relevant cases presented for this paper and their parameters, inputs and outputs are described in Section 4.

### 3.3. Experimental Setup and Model Particle System Characterization

The experimental setup includes a Z11-type tubular centrifuge designed by Carl Padberg Zentrifugenbau GmbH (CEPA, Lahr, Germany). All geometric boundaries used in the simulation tool are listed in Table 1 and predefined by the apparatus rotor itself. An eccentric screw conveyor (NETZSCH Pumpen und Systeme GmbH, Waldkraiburg, Germany) is able to transport a suspension with low pulsation and constant flow rate to the rotor inlet. For further information regarding the process layout for NP separation, the reader is referred to a preliminary study [31] which incorporated a similar setup.

To test and validate the adapted process model for 1D-PTDs, a non-spherical particle system is used in this study. The trade name Aerodisp^®^ W7512 S specifies a hydrophilic, fumed silicon dioxide (SiO${}_{2}$) suspension manufactured by Evonik Industries AG (Hanau, Germany). The product is synthesized in a flame hydrolysis process forming primary particles which then collide and partially attach to each other. This aggregation step is followed by an agglomeration of the non-spherical aggregates due to weak inter-particular interactions. Some examples showing the branched structure of the individual aggregates can be found here [29,77,78]. The SiO${}_{2}$ particles are well dispersed in demineralized water resulting in 30nm≤d≤130nm for the expected equivalent aggregate diameter. To ensure this dispersion state, the suspension is sonified (Ultrasound processor UP400St, Hielscher Ultrasonics, Teltow, Germany) prior to any classification experiment. The volume weighted, 1D-PTD was measured in a CPS 24,000 disk centrifuge (CPS Instruments Inc., Prairieville, LA, USA). The measuring principle of this analytical disk centrifuge (ADC) is based on an extinction screening at the outer edge of a rotating disk. A laser with wavelength 470 nm traverses through the transparent disk and sample at a small area before hitting the detector. Large particles reach this position earlier, successively followed by smaller particles. The advantage of the ADC measurement is that a density gradient in the disc effectively separates multiple size fractions, thereby reducing inter-particular forces by dilution and stabilizing the sedimentation during analysis. Using predefined procedures for each material, the raw extinction signal is translated into a 1D-PTD by combining Equation (6) and Mie scattering theory. Hence, only the equivalent diameter *d* and its distribution $q_{3,\mathrm{in}}\left(d\right)$ is measurable. This inevitably reduces the aggregates characterization to 1D. Nevertheless, referring to Figure 3 and Equation (25), the measured 1D-PTD of the RPS serves as an input for the 1D-TCM.

In order to assess the influence of particle concentration on the sedimentation behavior, the multisample analytical centrifuge (AC) LUMiSizer (LUM GmbH, Berlin, Germany) was utilized. The SiO${}_{2}$ suspension with a defined solids volume fraction was filled into a small cuvette and inserted onto a rotating platform. Their filling volume was 0.4 ml, spinning with a rotational speed of 4000 min${}^{-1}$ at a constant temperature of δ= 10 °C. Lowering δ in the measurement chamber helps to reduce particle diffusion. During sedimentation analysis a light source emits parallel rays of light [79] with the same wavelength as the ADC (λ=470 nm). While the rotation forms a meniscus in the perimeter of the cuvette, the AC measures an extinction profile along its entire height. Logging a new curve every 30 s provides a progression of sedimentation over time. The raw AC data can be used to calculate an apparent sedimentation coefficient distribution $g^{*}\left(s_{\mathrm{ext}}\right)$ either by a spatial or temporal differentiation of these extinction profiles [80,81]. In this study, the method published by Schuck et al. was used to derive $g^{*}\left(s_{\mathrm{ext}}\right)$ by means of least-squares boundary modeling (LSBM) [82]. The described procedure was first translated to a python code which automated extinction data input, pre-processing and $g^{*}\left(s_{\mathrm{ext}}\right)$ calculation. Note that the procedure neglects diffusion effects and involves an extinction weighting of the sedimentation coefficient. Unlike $s_{B}$ introduced in Section 2.2, the extinction weighted distribution $g^{*}\left(s_{\mathrm{ext}}\right)$ can not be used directly for the TCM model but is sufficient for an empirical approximation of the sedimentation hindrance coefficient *w* in Equation (11).

### 3.4. Virtual Particle System Creation and Characterization

**Figure 4:**
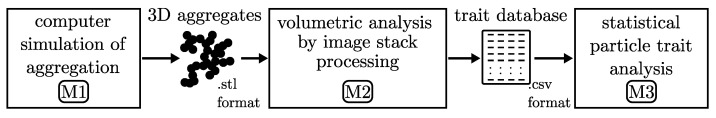
Schematic overview of the pipeline used to compute three dimensional (3D) particle trait distributions (PTDs) based on virtual silicon dioxide (SiO${}_{2}$) aggregates.

This section describes the workflow used to create a virtual SiO${}_{2}$ particle system (VPS) and compute an affiliated 3D-PTD. The entire method is structured in three sub-modules depicted in Figure 4.

The first module (M1) tackled the computer simulation of particle aggregation. From a general point of view, the formation of SiO${}_{2}$ aggregates during flame synthesis can be described by different kinetic growth models [83,84]. For the presented study, a custom diffusion-limited-aggregation (DLA) algorithm was used.

**Figure 5:**
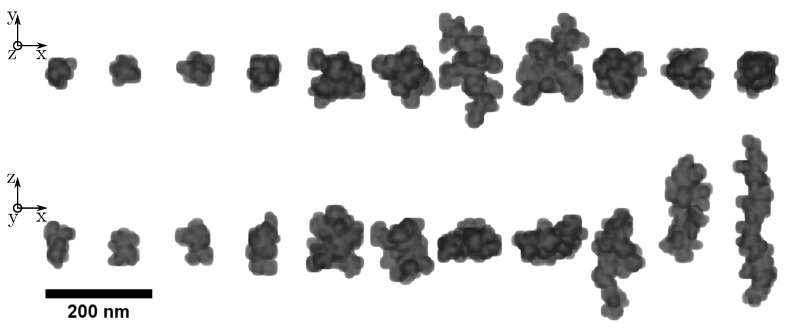
Two dimensional (2D) image of eleven unique nanoparticles (NPs) of the virtual particle system (VPS) created by the custom diffusion-limited-aggregation (DLA) algorithm (M1). Two different projections are shown to visualize differences is nanocluster form.

The computation starts with a seed particle in the center of a cubic grid. A second particle is released in the domain and moves step-wise to one of 26 possible lattice coordinates. If the randomized path intersects the initial seed perimeter, a collision is recorded. For a randomized total primary particle count, this process is repeated until a final 3D structure is build. Particles that wander outside the boundary are deleted and respawn [85,86]. A unique feature of the presented algorithm is that three individual spawn locations are defined. Usually, a particle trajectory starts at the surface of a sphere that surrounds the seed particle (option 1). Two additional options restrict the particles entry positions to the spheres equatorial circumference (option 2) and both polar regions (option 3). In each new run, the algorithm randomly chooses one option. This additional degree of randomization ensures that each particle has a unique structure with a lesser occurrence of compact aggregates. A more detailed and visual description of the custom DLA algorithm is provided as Supplementary Material of this manuscript. The setup was used to construct a collection of P=3771 aggregates for the VPS. Visual examples for some of the generated particles in the collection are shown in Figure 5.

The second module (M2) conducts a volumetric analysis of multiple 3D objects at once. Its functionality is inspired by quantitative computer tomography (CT) measurements of particles on the micro- and nanoscale [87]. In particle technology, nano- and micro-CT measurements are reconstructed into 3D images of a representative sample in a defined environment. Subsequently, particles in 2D image slices are segmented and counted. Each observed particle can be labeled with, for example, a vector of geometric properties. Detailed information on the state-of-the-art execution of CT-measurements and image processing workflows can be found here [88].

To recreate this methodology for the VPS, the open source content creation suite Blender [89] and its build-in Python application programming interface (API) was used to place roughly 100 particles out of the whole collection in a rectangular volume with a base area of 3.3 µm${}^{3}$. Thereby, care was taken that the individual particles did not overlap. At the end of this preparation procedure, 37 samples were exported in the Standard Triangle Language (STL) format and imported separately into the image processing software *Fiji* [90]. Afterwards, a plugin called *SciView* [91] created image stacks of each individual sample. An image resolution of 600×600 pixels results in a voxel size of 5.5 nm. At this point it should be mentioned that the technically feasible spatial resolutions in nano-CT are generally lower than the one applied in this module. To give an example, Ditscherlein et al. [88] reported applicable voxel sizes of 16 nm and 64 nm depending on the nano-CT measurement type. The applied spatial resolution in this study ensures an accurate recording and quantitative analysis of the nanocluster form. Processing the resulting binary image stacks in the *3D Objects Counter*-plugin [92] created an indexed voxel representation of every virtual sample. The obtained object maps were then analyzed by the well-known mathematical morphology analysis plugin *MorphoLibJ* [93]. Of particular interest is the automated determination of the 3D inertia ellipsoid and voxel volume. This volume in combination with a predefined solids density provides an approximation of the mass of each individual particle in the collection. As stated in Section 2, both particle volume and the form of a convex hull defined by the inertia ellipsoid are needed for sedimentation coefficient calculations. Therefore, the transverse and polar radii were used to calculate the particles elongation and flatness. Finally, the discrete particle information for $V_{P},f,e$ and *d* were compiled in a list with P=3771 entries.

The last module (M3) performs a statistical particle trait analysis based on a given input list of discrete information and outputs a user defined PTD. In accordance to Section 2.3 a 3D-PTD in discrete notation is a tensor of size (K×L×M). Each of the three variables *e*, *f* and *d* have user defined limits and discretize one dimension. During computation, an algorithm sorts each particle *n* with respect to their trait combination ${\left\{f,e,d\right\}}_{n}$ into a matching bin. Afterwards, the relative particle mass $m\left(f_{k},e_{l},d_{m}\right)$ in each class is calculated by adding up all particle volumes and multiply the sum class-wise with a predefined solids density $\rho_{p}$. In the final step, Equation (26) is used to compute the volume weighted 3D-PTD. This routine was applied to the VPS trait database output of M2 resulting in a multi-dimensional density distribution ${\tilde{q}}_{3,\mathrm{in}}\left(f_{k},e_{l},d_{m}\right)$ which served as one input for the 3D-TCM model.

The assignment of discrete property vectors based on high fidelity image analysis is a common practice in material science. For example, methods such as transmission (TEM) [94,95] or scanning electron microscopy (SEM) [96,97] are able to statistically characterize complex particle systems on a broad size range. In case of 3D image processing, Ditscherlein et al. [98] introduced an open access archive for particle-discrete CT. One major advantage of module M3 in the presented workflow is that it can process such database entries independently of the measurement methodology used. This means that, in future research, real-world particle data can be easily translated into a 3D-PTD and implemented into a 3D-TCM.

Modeling the fractionation of NPs is not the only application for particles emerging from the presented workflow. As an additional advantage, the universal data structures of the discrete particles leads to possible applications in other research areas. One example is the permeability investigation of porous media, which is largely determined by the spatial structure of the capillaries [99]. Similarly, numerical simulations on the performance of lithium ion batteries by Chauhan et al. can be highlighted in this regard. In their work, the authors generate idealized microstructures which mimic the spatial distribution of key materials such as carbon black and active materials in the cathode [100]. Here, the presented workflow of this study could offer an alternative to generate discrete 3D particles with variable microstructures. Consequently, the volumetric and statistical analysis (M2, M3) would add an additional factor to the performance simulation of lithium ion batteries: distributed particle features in more than one dimension.

## 4. Results

The presented results are divided into two parts. First, the experimental determination of the hindered settling coefficient is presented. Afterwards, a 1D- and 3D-TCM case output is shown. In the 1D setup, a 1D-PTD with 500 discrete bins for *d* serves as a density distribution input measured by an ADC. In the 3D case, a PTD with a total of 27,000 bins is utilized. For its construction, the VPS trait database provides all required information. Note that for reference and clarity all model outputs of the 3D case are marked with an accented tilde (e.g., $\tilde{T}$).

### 4.1. Hindered Settling Evaluation of Aerodisp^®^ W7512 S

**Figure 6:**
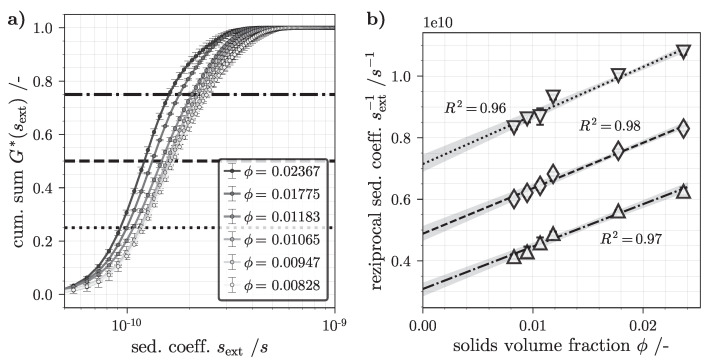
Cumulative sum distribution $G^{*}\left(s_{\mathrm{ext}}\right)$ derived by least-squares boundary modeling (LSBM) code based on analytical centrifuge (AC) extinction profiles (**a**). Reciprocal ext. weighted sedimentation coefficient $s_{25,\mathrm{ext}}^{-1}$, $s_{50,\mathrm{ext}}^{-1}$ and $s_{75,\mathrm{ext}}^{-1}$ plotted against ϕ of the analyzed silicon dioxide (SiO${}_{2}$) suspension; dotted, dashed and dash-dotted lines indicate a linear least squares fit (Equation (31)) with an associated coefficient of determination $R^{2}$ (**b**). Note that the lines indicate the connection between the data extracted from (**a**) and plotted in (**b**).

Following the steps described in Section 3.3, the RPS suspension was diluted with demineralized water and analyzed in the AC. Raw extinction data import and processing via LSBM leads to an apparent, extinction weighted sedimentation coefficient distribution. The advantage of this method is that the sedimentation does not need to be significantly advanced in order to derive a solution for $g^{*}\left(s_{\mathrm{ext}}\right)$, resulting in less time required per measurement. The evaluation set-point is chosen so that all particles are in motion, jet no complete separation of larger fractions takes place. By numerical integration of $g^{*}\left(s_{\mathrm{ext}}\right)$, the cumulative sum $G^{*}\left(s_{\mathrm{ext}}\right)$ was determined and plotted with its standard deviation in Figure 6a. Each curve is based on four analyzed samples and the mean value is plotted.

As described in Section 2.2, $s_{B}$ is a function of the sedimentation coefficient $s_{0,B}$ in infinite dilution, the solids volume fraction and the hindrance coefficient. In a subsequent step, the characteristic of the abscissa at $G^{*}\left(s_{25,\mathrm{ext}}\right)=0.25$, $G^{*}\left(s_{50,\mathrm{ext}}\right)=0.5$ and $G^{*}\left(s_{75,\mathrm{ext}}\right)=0.75$ were determined and plotted reciprocally against the solids volume fraction in Figure 6b. In other words, the intersections between each of the horizontal lines and the $G^{*}\left(s_{\mathrm{ext}}\right)$ cumulative sum distribution in Figure 6a lead to the 18 plotted datapoints shown in Figure 6b. Assuming the linear dependence,

(31) $$\frac{1}{s_{\mathrm{ext}}}=\frac{w}{s_{0,\mathrm{ext}}}\phi +\frac{1}{s_{0,\mathrm{ext}}},$$

after rearranging Equation (11), a least squares fit yields *w* and $s_{0,\mathrm{ext}}$ as functional parameters [101,102]. A parallel evaluation for all three distribution reference points leads to a mean hindered settling coefficient of w=32.44±9.40 for the SiO${}_{2}$ suspension. The uncertainties in *w* can be explained by error propagation. The averaging of four individual measurements from the LSBM leads to noticeable slope deviations marked as gray areas beneath the linear fit curves in Figure 6b. Nevertheless, the AC analysis provides a correction for the sedimentation conditions based on inter-particle interactions, which are enhanced with an increased solids volume fraction. Therefore it is used in both the 1D- and 3D-TCM for sedimentation hindrance modeling.

### 4.2. TCM Results for 1D Classification

**Figure 7:**
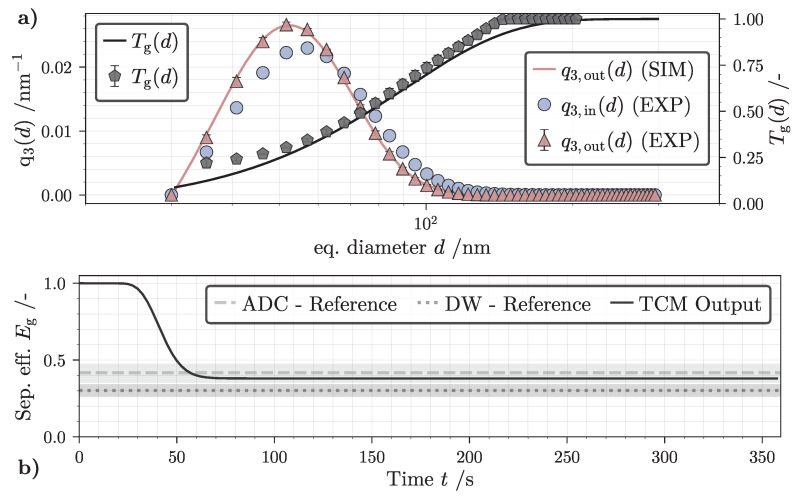
(**a**) Volume weighted density distribution of feed and centrate suspension (left ordinate) and global grade efficiency (right ordinate) at $t_{N}=360\;s$. Markers indicate experimental data acquired by analytical disk centrifuge (ADC) measurements, solid lines mark the tubular centrifuge model (TCM) case output. (**b**) Global separation efficiency *versus* time calculated by the TCM model. Experimentally acquired reference with two independent methods drawn with horizontal dashed and dotted lines, gray areas highlight the standard deviation of both measurements.

This part illustrates a 1D classification experiment of the RPS used to validate the 1D-TCM. Utilizing the experimental setup and parameters highlighted in Section 3.3 and Table 1, the analyzed 1D-PTDs of both feed suspension and centrate are shown in Figure 7a. Additionally, ADC measurements yield an experimental value for the global separation efficiency $E_{g}$ used to calculate a 1D graph of $T_{g}$ (Equations (28)–(30)). In general, experimentally determined values are shown as points whereas the 1D-TCM simulation output is marked as solid lines. The separation experiment was performed twice and both ADC and gravimetric analysis were performed in triplicate to derive the mean and standard deviation for $\phi_{\mathrm{in}}$, $\phi_{\mathrm{out}}$ and $T_{g}$. Results for both $q_{3,\mathrm{out}}$ and grade efficiency are in good agreement with comparable classification scenarios found in the literature [27].

Continuing with the validation procedure, identical boundary conditions and the acquired 1D-PTD input $q_{3,\mathrm{in}}\left(d\right)$ supply a working 1D-TCM case as proposed schematically in Figure 3. The total simulation time $t_{N}$ is limited to 360 s to match the suspension sampling time in the experimental study. Following a preliminary evaluation procedure described in Appendix A.1, the spatial discretization is set to J=20 compartments and the simulation timestep is set to Δt=0.5s.

Figure 7b highlights the history of $E_{g}$ (solid line) as well as the experimental reference acquired by differential weighing (DW) and ADC measurements. The initial model output indicates a separation efficiency of one since the rotor is pre-filled with demineralized water. After 30 seconds, the suspension and sediment zone gradually populate with solid material due to particle entrance and deposition. After roughly one minute, the process runs practically stationary with almost no change in $E_{g}$. This is explained by the low feed concentration of the SiO${}_{2}$ suspension resulting in no significant sediment buildup and effect on sedimentation behavior. Likewise, the influence of sedimentation hindrance is barely noticeable since measured uncertainties in *w* result in a 0.48% fluctuation of the simulated mean cut size $d_{T,50}$. This conclusion surfaced after a sensitivity analysis in which *w* was adjusted multiple times within the scope of its standard deviation. This is in accordance to experimental results conducted by Spelter et al. [29] describing a negligible effect of sedimentation hindrance for $\phi_{\mathrm{in}}\leq 0.5\;v\%$ for the identical particle system. In summary, numerical simulation results and experimental data are in a very good agreement. Most discernible deviations can be explained by a very low concentration of NPs at the beginning (d≥ 150 nm) and at the end (d≤ 35 nm) of a 1D-PTD analysis with the ADC. Overall, these findings underline the validity of the current results in case of the 1D-TCM model. Chances made to the initial dynamic process model introduced by Gleiss [72] did not affect the congruence of experiment and simulation.

The presented TCM model version is tailored to flexible, multi-dimensional PTD inputs and short simulation times. This means that if long-term experiments are to be simulated, model accuracy could be worse. The importance of sediment build-up consideration has already been proven experimentally by several authors [29,103]. Therefore, Gleiss et al. [37] and Menesklou et al. [39] implemented an adequate method building upon empirical measurements for the consolidation mechanism in decanter centrifuges. For now, the need for long-term model accuracy is secondary to the successful implementation of 3D-PTD into the TCM model. Future research, however, needs to incorporate polydisperse, multi-component sediment build up and consolidation with adequate material functions. Nonetheless, in the presented work, a high model accuracy was achieved for the numerical simulation of 1D classification in tubular centrifuges.

### 4.3. Visualization of 3D-PTDs

**Figure 8:**
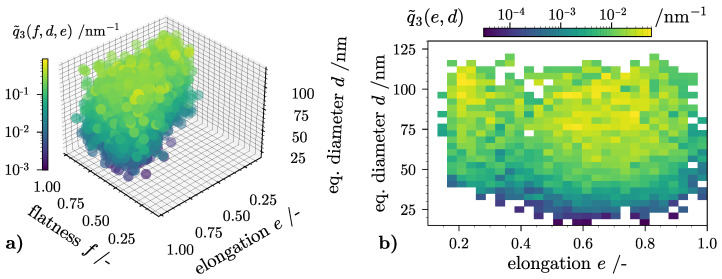

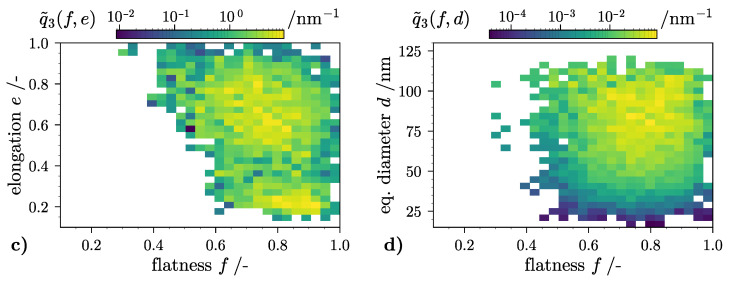
(**a**) Three dimensional (3D) particle trait distribution (PTD) ${\tilde{q}}_{3,\mathrm{in}}\left(f,e,d\right)$ of virtual silicon dioxide (SiO${}_{2}$) particle system: discretization over the three distinct traits d, e and f; (**b**–**d**) Marginalized distributions ${\tilde{q}}_{3,\mathrm{in}}$ visualized in a two dimensional (2D) grid.

The pipeline output presented in Section 3.4 is best described as a 3D array that holds the PTD information. Unlike 1D-PTDs usually found in particle technology, their graphical visualization is more challenging. Figure 8a shows the computed PTD of the VPS. Here, discretization settings with K=L=M=30 nets 27,000 individual trait classes. Data presentation resembles a cloud where each individual bin is color in accordance to a certain probability density ${\tilde{q}}_{3}\left(f,e,d\right)$. High values (yellow color) indicate that particles in this trait class are more likely to be found during a quantitative sample analysis of the whole collection. Empty bins are rendered as transparent volumes. The color-map on the left indicates the volume weighed density distribution. An advantage of this visualization method is a fast identification of elongated, flat or bladed NP populations. In the given example, both compact particles with e.g., f=e≥0.9 and elongated aggregates with e≤0.3 and f≥0.8 are present in the sample and can be detected with the naked eye. A severe disadvantage poses the noticeable cluttering of density distribution data. Unless the 3D plot is viewed from multiple angles, parts of the distribution are obscured. At the same time, the interior data are not visible at all. As described in Section 2.3, one possible solution is distribution marginalization. As an example, Figure 8c shows the 2D-PTD obtained by Equation (15). In this graphical representation of the VPS, one can identify the presence of both elongated and more compact aggregates in the range of 30nm≤d≤120nm. Hence, in combination with the other two marginalization options ${\tilde{q}}_{3,\mathrm{in}}\left(f,d\right)$ and ${\tilde{q}}_{3,\mathrm{in}}\left(e,d\right)$, the discussion of discrete, higher dimension probability data is simplified.

**Figure A2:**
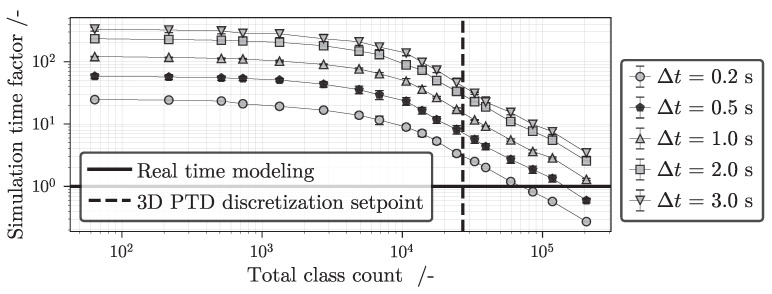
Simulation time factor in dependence of the total distribution class count for different time step sizes. Unity indicates a real-time measurement. Values greater than one factorize the simulation speed increase.

Another point to address is the distribution resolution or in parameter expression, the total discrete class count. One negative aspect of a high PTD resolution is linked to an elevated computational effort in the 3D-TCM (see Figure A2). However, a low resolution with a total class size below 100 inevitably leads to higher discretization errors, since detailed particle trait information is lost. A preliminary study justifying the discretization settings used for the 3D-TCM case is given in Appendix A.2.

### 4.4. TCM Results for 3D Fractionation

**Figure 9:**
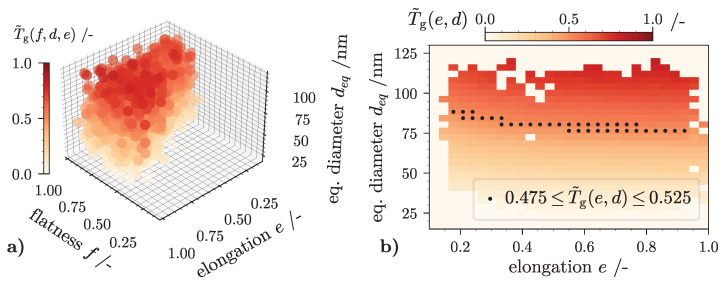
(**a**) Three dimensional (3D) grade efficiency ${\tilde{T}}_{g}\left(f,e,d\right)$ of virtual silicon dioxide (SiO${}_{2}$) particle system after separation: discretization over the three distinct traits d, e and f; (**b**) Marginalized data over particle traits *e* and *d*.

This section covers the 3D-TCM case output for the VPS. The simulation domain is identical to the presented 1D process simulations. This means that all boundary settings mentioned in Section 4.2 stay fixed. The simulation time is set to 360 s, the total compartment count is J=20 and the time step size equals 0.5 s. Additionally, the empirical hindered settling coefficient (see Section 4.1) is reapplied. The 3D-TCM output results are outlined in two figures: The presentation in Figure 9 is identical to the one in Figure 8, since both ${\tilde{T}}_{g}\left(f,e,d,t=360\right)$ and ${\tilde{q}}_{3,\mathrm{out}}\left(f,e,d\right)$ share the same data structure and array shape. The 3D grade efficiency illustration, acquired by Equation (29), implies the same negative aspects regarding readability on 2D prints. Nevertheless, it can be seen that particle classes with larger values for *d* tend to be separated more effectively in the centrifuge. This is indicated by a more saturated coloration of the individual classes. Since the equivalent diameter reflects the particles volume (Equation (1)) which dominantly impacts particle sedimentation rates according to Equation (4), this observation is expected. The same trend can be noticed in Figure 9b. Here, the grade efficiency,

(32) $$\begin{array}{cc} {\tilde{T}}_{g}\left(e_{l},d_{m},360\;s\right) & =1-\frac{{\tilde{E}}_{g}\left(360\;s\right)\;{\tilde{q}}_{3,\mathrm{out}}\left(e_{l},d_{m},360\;s\right)}{{\tilde{q}}_{3,\mathrm{in}}\left(e_{l},d_{m}\right)}, \end{array}$$

is calculated from the marginalized PTD of the feed and centrate suspension, isolating the traits elongation and particle volume. A marginalization to 2D draws attention to the fact that SiO${}_{2}$ grade efficiencies are slightly larger for more elongated particles (e→0). For a guide to the eye, classes that hold a value of $0.475\leq {\tilde{T}}_{g}\left(e_{l},d_{m},360\;s\right)\leq 0.525$ are marked with a single black dot.

**Figure 10:**
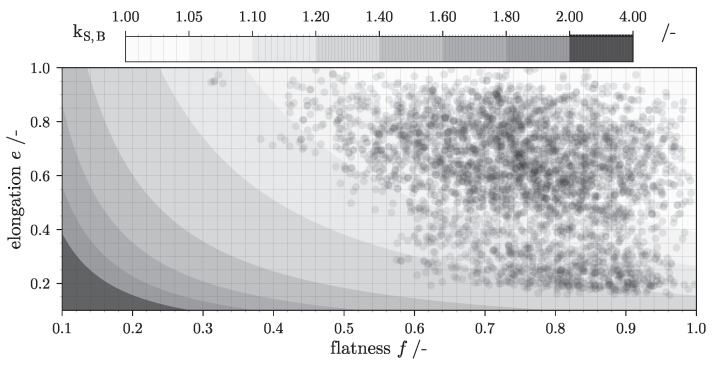
Heatmap of drag correction factor $k_{S,B}$ for ellipsoids *versus* their elongation and flatness based on Equation (9) found in [53].

Revisiting the presented form analysis (Section 3.4) of all 3D aggregates which populate the VPS can explain these observations. Figure 10 shows $k_{S,B}$-values in dependence of elongation and flatness calculated with Equation (9). Additionally, for each individual particle, P=3771 black dots are plotted. Their position is set according to their *e* and *f* values which were recorded by the volumetric analysis of M2. Looking at the spatial distribution of all dots, only a small minority of the particles generated by the custom DLA algorithm can be associated with $k_{S,B}$-values greater than 1.2. Consequently, the sedimentation coefficient (Equation (10)) for a majority of the particles is similar to that of a sphere with equal volume. As a result, the overall impact of particle form on the model output is almost negligible. Despite this fact, it should be positively emphasized that the separation of several different form fractions can be identified with this method. To give an example, one could imagine a particle collective of small, compact and larger rod-like particles. Due to their differences in elongation, their fractions are easily identified in a 3D-PTD. Referring to the presented output of any 3D-TCM model, their separation efficiency can be calculated and visualized intuitively with the presented methodology. In summary, it can be said that the integration of Equation (10) into the TCM mass balance calculation is functional. Both volume and particle form, represented by a surrounding inertia ellipsoid with elongation *e* and flatness *f*, is considered in the calculation of the sedimentation coefficient. The simulation-tool is able to visualize the outcome of a tubular centrifuge separation experiment in more than just one dimension even for small deviations in particle form.

**Figure 11:**
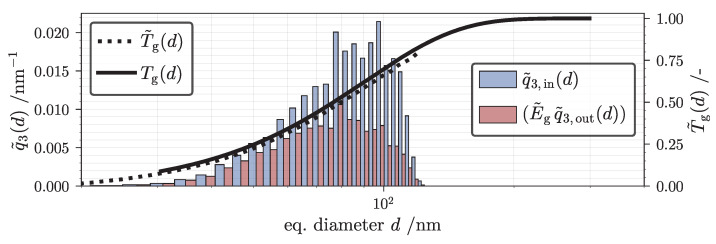
Volume weighted density distribution of virtual particle system (VPS) feed- and centrate suspension (left ordinate) and global grade efficiency (right ordinate). Distribution data are acquired by a marginalization of three dimensional (3D) tubular centrifuge model (TCM) output. Dimensional reduction applied for comparison between $T_{g}$ and ${\tilde{T}}_{g}$.

Closing the gap between the 3D-TCM and a traditional separation process outcome, the marginalized 1D-PTD and the associated grade efficiency curve is shown in Figure 11. A bar-plot is used to view the 30 classes which discretize the equivalent diameter. Blue bars and their height reference the inlet PTD whereas red bars show the product of ${\tilde{E}}_{g}$ and the marginalized outlet PTD. One important question to address is whether the global grade efficiency in the 3D case is comparable to the preceding RPS classification experiment. This should be the case since both the RPS and VPS share the same solid density and a similar equivalent diameter range. To compare both conducted TCM cases, the 1D global grade efficiencies $T_{g}$ and ${\tilde{T}}_{g}$ are added as line plots to Figure 11. As expected, the data are congruent with a slight deviation for higher particle volumes. This outcome can be explained by the presence of elongated particles with d≥80nm in the collection. For a visual reference, attention is drawn to the upper left quadrant of ${\tilde{q}}_{3,\mathrm{in}}\left(d\right)$ in Figure 8b and again to Figure 10. According to the fundamentals of form dependent NP settling, these discrete classes have trait combinations which lead a lower value for $s_{B}$.

Hence, during iterative short-cut computation of $T_{j}\left(s_{B}\right)$, these mass fractions are separated less effectively. In the 1D setup, however, discrete sedimentation rates are higher due to the strict limitation of $k_{S,B}=1$. As a result, the mean cut size is shifted slightly towards smaller particles. Since the VPS is considered a unique particle system, there are no comparable experimental or model results present in the literature. Hence, only the plausibility of the results are validated in this study. A detailed validation of 3D grade efficiencies will demand precise and adapted methodologies regarding the multi-dimensional analysis of NP properties used in future research. In light of this, the presented work establishes an adaptable routine for the creation and detailed trait analysis of NP collectives. Consequently, the proposed workflow and working adjustments made to the TCM facilitate these future efforts to conduct an experimental validation in multi-dimensional fractionation.

The results imply that the novel simulation routine is capable of addressing the NP form fractionation in tubular bowl centrifuges at a fixed set of boundary condition. However, the added benefit of the adapted multi-dimensional TCM does not end here. Model parameters such as fluid viscosity, angular velocity and volumetric flow rate can be varied with little effort resulting in an updated separation result. A completely automated adjustment of these boundary conditions is also conceivable and can be implemented by only a few changes in the program code. This is an important prerequisite for the efficient performance of parameter studies that can quantify the influence of individual process parameters. Since the novel model also calculates and displays 3D separation efficiency data (see Figure 9), optimization with respect to one or more particle features is possible. Lastly, the feed material can be easily modified, either based on real-world or computer-generated particles derived from simulated (see Section 3.4 and Figure 4) or real-world discrete particle databases [98].

## 5. Conclusions

Traditionally, the separation efficiency in tubular bowl centrifuges is evaluated as a function of particle size, represented by the radius of a volume equivalent sphere. However, current research draws attention to advantageous product properties linked to, for example, the form distribution of a particle collective. Therefore, this work develops a simulation tool for fractionation modeling of multi-dimensional NP systems in tubular bowl centrifuges. To facilitate the numerical calculation of both one- and multi-dimensional grade efficiencies, an existing model by Gleiss [72] is modified. In its core, the dynamic model solves mass balance equations for a finite number of compartments that spatially discretize the sedimentation zone and define the solids residence time.

Investigations first addressed the 1D classification of a real-world SiO${}_{2}$ nanosuspension. In a direct comparison, numerical simulations predicted the separation outcome with very high accuracy. In both the 1D-TCM and reference measurements for the achievable grade efficiency only the equivalent diameter was considered a particle trait.

For a second simulation case, 3771 virtual SiO${}_{2}$ aggregates were created by a custom DLA algorithm and characterized individually by direct image analysis. Their form and volume is discretized by three variables: elongation, flatness and equivalent diameter. By means of an automated statistical trait analysis, a volume weighted PTD of the particle system is calculated which served as the new TCM model input in a 3D fractionation setup. Here, form-dependent settling was implemented by empirical correlations found in the literature. Numerical simulations confirm that particle form has a much smaller influence on their sedimentation rate compared to particle volume. The presented discussion implies that the separation result per particle trait class is only influenced by a more pronounced elongation or flatness $\left(e<<1,f<<1\right)$. With regard to the precise adjustment of the product properties through, for example, modifications to the form distribution, a major challenge in multi-dimensional fractionation emerges. The majority of these separation tasks are extremely difficult in a singular centrifugal field of a tubular bowl centrifuge. This is because similarities in the sedimentation rate occur frequently even for a broad range of non-spherical particles. Future approaches could include the addition of a second force field, whose parameters show a stronger correlation regarding the NPs elongation and flatness. Adjustments of the tube centrifuge geometry are also conceivable, which can be studied and optimized with the presented dynamic simulation tool focusing on the apparatus performance in multi-dimensional fractionation.

Nevertheless, added value by the presented TCM emerges from a facilitated communication on multi-dimensional grade efficiencies for tubular bowl centrifugation processes. Moreover, the proposed algorithm that computes 3D-PTDs is applicable to any discrete dataset of particle traits which represent physical properties of nanosuspensions. This includes statistical data acquired from, e.g., micro-CT and TEM image analysis. Consequently, the workflow enables a fast and flexible numerical assessment of separation processes for a variety of different nanosuspensions. Furthermore, advancements in multi-dimensional characterization of particulate products in separation technology will assist with future model validation attempts. Lastly, real-time computation speeds of the 3D-TCM highlight its potential use in model predictive control scenarios. The novelty here comes with the simultaneous consideration of two or more separation criteria in process optimization.

## Figures and Tables

**Table 1:** Overview of the centrifuge geometry, fluid and particle system properties.

Parameter	Symbol	Value	SI unit
weir radius	$r_{w}$	0.0073	m
wall radius	$r_{b}\left(t_{n=0}\right)$	0.0215	m
rotor length	*L*	0.175	m
angular velocity	ω	40,000	$s^{-1}$
flow rate	${\dot{V}}_{f}$	$5\times 10^{-6}$	$m^{3}s^{-1}$
solids density	$\rho_{p}$	2200.0	$\mathrm{kg}\;m^{3}$
mean particle size	$d_{50}$	64.39	nm
solids volume fraction	$\phi_{\mathrm{in}}$	$3.0\times 10^{-4}$	−
liquid density	$\rho_{l}$	998.207	$\mathrm{kg}\;m^{3}$
liquid viscosity	$\eta_{l}$	$1.0027\times 10^{-3}$	$\mathrm{kg}\;m^{-1}\;s^{-1}$

**Figure A1:**
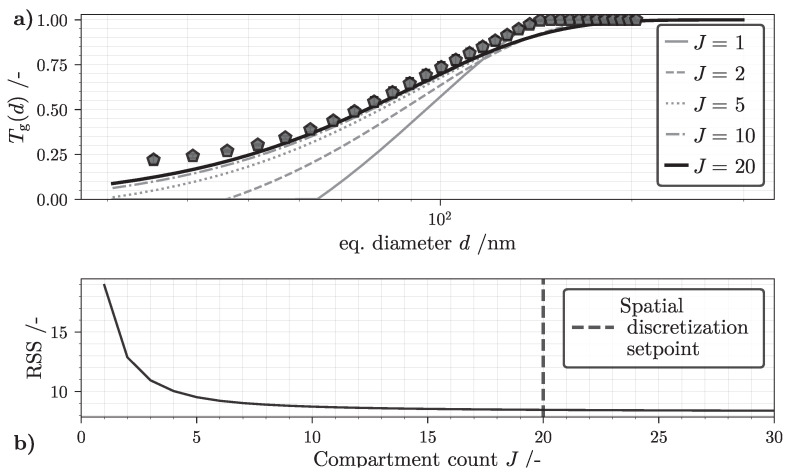
(**a**) Global grade efficiency for the RPS classification with boundary conditions listed in Table 1; markers show the ADC reference measurement and solid lines show the 1D-TCM output for different spatial discretization settings; (**b**) RSS calculated with Equation (A1) for different spatial discretization settings; the dashed line indicates the setpoint used for all simulations shown in the main manuscript.

## Data Availability

The data presented in this study are available on request from the corresponding author.

## Supplementary Materials

The following supporting information can be downloaded at: https://www.mdpi.com/article/10.3390/nano12183161/s1, Figure S1: Schematic illustration of a 2D DLA algorithm. Steps and principles of aggregate formation are showcased in 2D but are easily transferable to the 3D setup used in this work; Figure S2. Visualization of different 3D spawn regions for the custom DLA algorithm: a) equatorial, b) sphere, c) polar; Table S1. Parameters used in the custom DLA algorithm.

## Abbreviations

The following abbreviations are used in this manuscript:

1D	one dimensional
2D	two dimensional
3D	three dimensional
AC	analytical centrifuge
ADC	analytical disk centrifuge
API	application programming interface
CT	computer tomography
DLA	diffusion-limited-aggregation
DW	differential weighting
EXP	experiment
IMVM	Institute of Mechanical Process Engineering and Mechanics
KIT	Karlsruhe Intitute of Technology
LSBM	least-squares boundary modeling
NP	nanoparticle
PSD	particle size distribution
PTD	particle trait distribution
RPS	real-world particle system
SEM	scanning electron microscopy
SIM	simulation
SiO${}_{2}$	silicon dioxide
TCM	tubular centrifuge model
TEM	transmission electron microscopy
VPS	virtual particle system

## Appendix A. TCM Parameter Selection

### Appendix A.1. Spatial Discretization

The total compartment count *J* defines the spatial discretization of the separation zone. Therefore, the total number of equations solved by the TCM algorithm (Figure 3) is greater for higher resolutions. This has a negative effect on the overall computation time required. In an effort to choose an optimal value for *J*, the classification reference experiment was used. In Figure A1a gray pentagonal markers show reference values for $T_{g,\mathrm{ADC}}$ acquired by ADC measurements of feed and centrate samples after separation. The plotted lines indicate the simulated 1D-TCM output for the global grade efficiency $T_{g,\mathrm{TCM}}$ for different values of *J*. All other parameters were kept constant.

Further on, the residual sum of squares,

(A1) $$\begin{array}{c} RSS=\sum_{m=1}^{M}{\left(T_{g,\mathrm{TCM}}\left(d_{m}\right)-T_{g,\mathrm{ADC}}\left(d_{m}\right)\right)}^{2}, \end{array}$$

between TCM data and ADC reference data was calculated. Higher values indicate an overall greater deviation between the two datasets. Plotted over the compartment count in Figure A1b, the RSS value provides a numerical output for the simulation accuracy over a broad range of spatial discretization options. The data suggests that a good compromise between computational effort and accuracy can be achieved when *J* is set to 20. Each TCM model output shown in the main manuscript are based on this spatial resolution setup.

### Appendix A.2. Temporal Discretization

One of the most predominant advantages of tubular centrifuge flow-sheet simulation using short-cut models to describe particle separation is a reduced computational effort. However, the resolution of PTD discretization plays an important role when aiming at real-time simulation speeds. In 1D, the total class count is usually dictated by the measurement methodology and data post processing. In this work, the ADC measurement produces a 1D-PSD $q_{3,\mathrm{in}}$ with 500 bins. By adding two additional particle traits the same bin size is extended by the power of three which leads to an increase in computation time. To quantify this elevation, the total simulation time for different total class counts is recorded and analyzed. The results are highlighted in Figure A2.

All simulations were performed on a personal computer with an Intel^®^ Core^™^ i5-7400 CPU (Santa Clara, CA, USA) running at 3.0 GHz with 15.9 GB of RAM. This also includes all the results shown in Section 4. Data points shown in Figure A2 are mean values of multiple simulation runs under different system loads of the processor and memory. Consequently, a small standard deviation in the simulation time factor can be seen. The results indicate that for class sizes below $10^{4}$ the TCM model can preemptively calculate one separation experiment at least 100 times faster. This is true even for the smallest observed simulation time step size of Δt=0.2. For a higher number of trait classes, the simulation is slowed down significantly. The above mentioned arithmetic operation of repeated tensor multiplications and summations (Equations (20) and (24)) can be identified as the main bottleneck. However, the TCM code optimizes these operations uing *numpy*-arrays as the main data structure for n-dimensional arrays and build-in functions of the *numpy* package as well. With this in mind, a total class count of $2.7\times 10^{3}$ is chosen for the 3D case. This input setting offers a high resolution in the presented 3D-PTD and global grade efficiencies for an appropriate communication of the separation experiment outcome with a reasonable degree of discretization error.
