# Supplementary material for: Real-Time Modeling of Volume and Form Dependent Nanoparticle Fractionation in Tubular Centrifuges
Source: Nanomaterials (Basel). 2022 Sep 12;12(18):3161. doi: 10.3390/nano12183161 (PMC9500975; doi:10.3390/nano12183161)
Supplement: Supplementary file 1 [file nanomaterials-12-03161-s001.zip › mdpi_nanomaterials_winkler2022_sm.pdf]

Real-Time Modeling of Volume and Form Dependent Nanoparticle Fractionation in Tubular Centrifuges: Supplementary Material

Marvin Winkler[1], Frank Rhein[1], Hermann Nirschl[1], and Marco Gleiss[1]

[1]**Institute of Mechanical Process Engineering and Mechanics, Karlsruhe Institute of Technology (KIT)**

ABSTRACT

A dynamic process model for the simulation of nanoparticle fractionation in tubular centrifuges is presented. Established state-of-the-art methods are further developed to incorporate multi-dimensional particle properties (traits). The separation outcome is quantified based on a discrete distribution of particle volume, elongation and flatness. The simulation algorithm solves a mass balance between interconnected compartments which represent the separation zone. Grade efficiencies are calculated by a short-cut model involving material functions and higher dimensional particle trait distributions. For the one dimensional classification of fumed silica nanoparticles, the numerical solution is validated experimentally. A creation and characterization of a virtual particle system provides an additional three dimensional input dataset. Following a three dimensional fractionation case study, the tubular centrifuge model underlines the fact that a precise fractionation according to particle form is extremely difficult. In light of this, the paper discusses particle elongation and flatness as impacting traits during fractionation in tubular centrifuges. Furthermore, communications on separation performance and outcome are possible and facilitated by the three dimensional visualization of grade efficiency data. Future research in nanoparticle characterization will further enhance the models use in real-time separation process simulation.

Keywords: solid-liquid separation; fractionation; tubular centrifuges; dynamic modeling; real-time simulation, multi-dimensional particle properties

1 COMPUTER SIMULATION OF AGGREGATION: CUSTOM DLA ALGORITHM

In the main manuscript, a workflow is presented which enables the generation of three dimensional (3D) particle trait distributions (PTDs) based on virtually generated particles. Here, three different modules work in conjunction to compute the desired output.
As supplementary material for the main part, the functionality of the first module is described in more detail. Its task is the computer simulation of nanoparticle (NP) aggregation. For this, a custom diffusion-limited-aggregation (DLA) algorithm is used. Figure S1 shows a schematic of all processes during particle creation. In the following, the basic algorithm is first explained for two dimensions (2D). Afterwards, the main differences between a 2D and 3D setup is highlighted.

The computation starts with the initialization of a seed particle in the middle of a rectangular grid of size $\left(N_{\text{grid}}^3\right)$. The second step introduces a second particle in a well defined, circular spawn perimeter around the seed. This particle, however, is able to move in a total of eight directions. The particles trajectory or path is randomized with a defined number of steps N_{step}. Exemplified trajectories are shown in Figure S1. If this path intersects the direct perimeter of the seed particle, a collision is registered and the aggregate grows. This process is repeated with a set amount N_{p} of primary particles. All positions where particles can attach to the formed aggregate are denoted as *reactive* grid points. As the maximum circular aggregate radius r_{agg} increases, the spawn radius r_{s} is also dynamically adjusted. Particles which travel further than the outer perimeter are reintroduces to the grid. Furthermore, if a particles path ends without any intersection with the reactive grid, a new path is calculated from its last position.

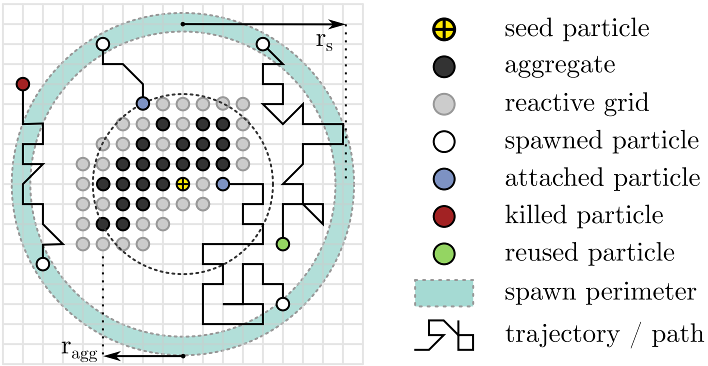

Figure S1. Schematic illustration of a 2D DLA algorithm. Steps and principles of aggregate formation are showcased in 2D but are easily transferable to the 3D setup used in this work.

This concept can be easily transferred to a 3D grid where newly introduced particles now have 26 possible directions in which they can move. A special feature of the procedure presented in this work is the spawn perimeter adjustment. When building multiple aggregates in succession, the algorithm can choose randomly between three options. The first option draws a circle around the seed particle with radius r_{s} (see Figure S2b). This way, particles are allowed to start their random path at any given point of the spheres surface. The second option limits this area to the equatorial circumference of the same sphere (see Figure S2a). Lastly, if the algorithm chooses the third option, the area is further decreased to the polar regions of the spherical boundary (see Figure S2c)

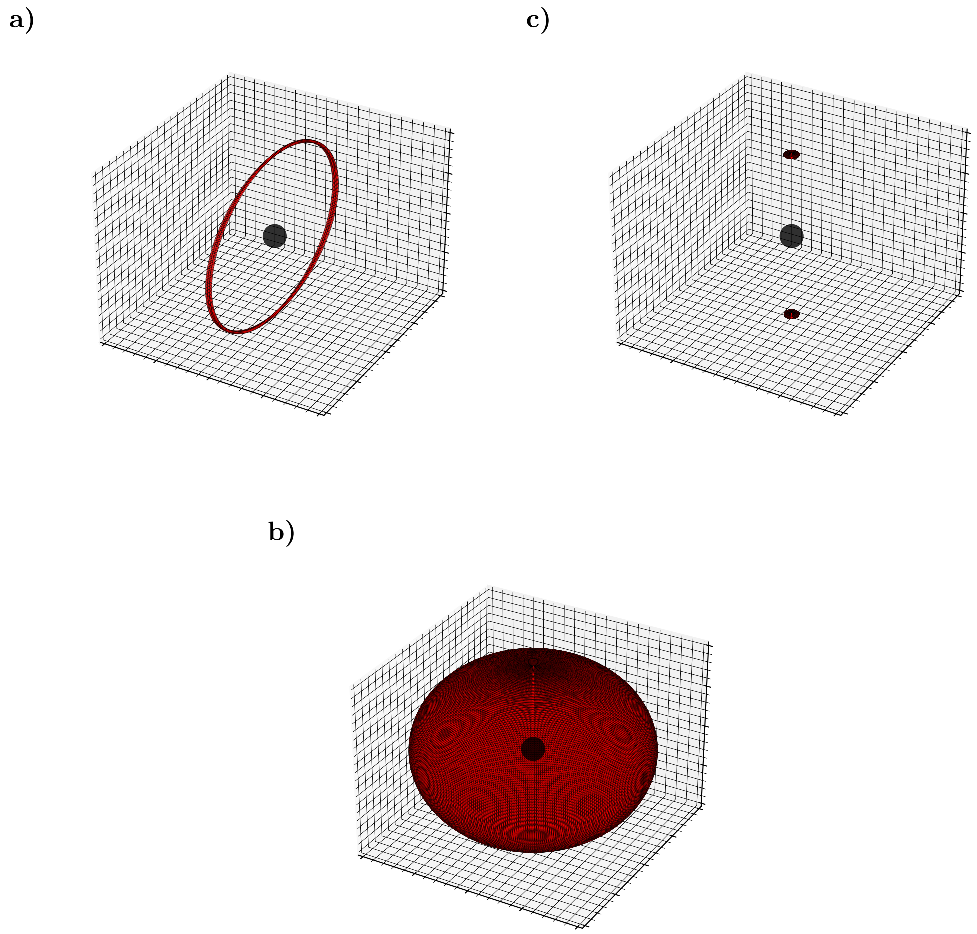

Figure S2. Visualization of different 3D spawn regions for the custom DLA algorithm: a) equatorial, b) sphere, c) polar.

As a result of the spawn perimeter reduction it can be observed that created aggregates are either more elongated (polar spawn option), flat (equatorial spawn option) or compact (spherical spawn option). Visual examples for the impact of this additional feature in the DLA code are highlighted in Figure 5 in the main manuscript.
The final volume of each individual aggregate is influenced by the number of primary particles released during the calculation. For every instance, a random number between 20 and 500 was set. An overview of all parameters used to create 3771 individual particles for the virtual particle system (VPS) are listed in Table S1.

Table S1. Parameters used in the custom DLA algorithm.

Parameter	Value	SI unit
grid size N_{grid}	120	–
rectangular grid size dimensions	$120 \times 120 \times 120$	–
minimal primary particle count $N_{p,min}$	20	–
maximum primary particle count $N_{p,max}$	500	–
path step size N_{step}	1000	–
